# The role of TRPV1 in different subtypes of dorsal root ganglion neurons in rat chronic inflammatory nociception induced by complete Freund's adjuvant

**DOI:** 10.1186/1744-8069-4-61

**Published:** 2008-12-04

**Authors:** Lu Yu, Fei Yang, Hao Luo, Feng-Yu Liu, Ji-Sheng Han, Guo-Gang Xing, You Wan

**Affiliations:** 1 Neuroscience Research Institute, School of Basic Medical Sciences, Peking University, Beijing, PR China; 2 Department of Neurobiology, School of Basic Medical Sciences, Peking University, Beijing, PR China; 3 Key Laboratory for Neuroscience, the Ministry of Education/the Ministry of Public Health. 38 Xueyuan Road, Beijing 100191, PR China

## Abstract

**Background:**

The present study aims to investigate the role of transient receptor potential vanilloid 1 (TRPV1) in dorsal root ganglion (DRG) neurons in chronic pain including thermal hyperalgesia and mechanical allodynia. Chronic inflammatory nociception of rats was produced by intraplantar injection of complete Freund's adjuvant (CFA) and data was collected until day 28 following injection.

**Results:**

Thermal hyperalgesia was evident from day 1 to day 28 with peak at day 7, while mechanical allodynia persisted from day 1 to day 14 and was greatest at day 7. Intrathecal administration of AMG 9810 at day 7, a selective TRPV1 antagonist, significantly reduced thermal hyperalgesia and mechanical allodynia. TRPV1 expression in DRG detected by Western blotting was increased relative to baseline throughout the observation period. Double labeling of TRPV1 with neuronal marker neurofilament 200 (NF200), calcitonin gene-related peptide (CGRP) or isolectin B4 (IB4) was used to distinguish different subtypes of DRG neurons. TRPV1 expression was increased in the medium-sized myelinated A fiber (NF200 positive) neurons and in small non-peptidergic (IB4 positive) neurons from day 1 to day 14 and was increased in small peptidergic (CGRP positive) neurons from day 1 to day 28.

**Conclusion:**

TRPV1 expression increases in all three types of DRG neurons after CFA injection and plays a role in CFA-induced chronic inflammatory pain including thermal hyperalgesia and mechanical allodynia.

## Background

Transient receptor potential vanilloid 1 (TRPV1) has been shown to be a ligand-gated non-selective cation channel following its successful cloning from rat [1] and human [2,3] sensory neurons. It can be activated by capsaicin and resiniferatoxin [4], as well as noxious heat [1], low pH [5], some lipid mediators such as anandamide [6] and lipoxygenase product 12-(*S*) hydroxyeicosatetraenoic acid [7]. Results from mutant mice lacking TRPV1 demonstrate that TRPV1 is essential for hyperalgesia induced by either acid or heat [8,9].

TRPV1 has been widely studied in acute inflammatory nociception [1,10,11]., but recently, the role of peripheral TRPV1 in chronic inflammatory pain has begun to attract more interest. For acute inflammatory pain, TRPV1 expression has been shown to be increased in hind paw skin, sciatic nerve and DRG 2 and 7 days following induction of inflammatory pain with complete Freund's adjuvant (CFA) injection [12,13]. Oral or intrathecal administration of TRPV1 antagonists A-784168 and A-795614 have been shown to reduce CFA-induced thermal hyperalgesia and mechanical allodynia [14], application of (*E*)-3-(4-*t*-butylphenyl)-*N*-(2,3-dihydrobenzo [*b*][1,4] dioxin-6-yl)acrylamide (AMG 9810), the first cinnamide TRPV1 antagonist, has been shown to block capsaicin-induced nociceptive behaviors like eye wiping and to reverse inflammatory thermal hyperalgesia in rats [15]. To address the role of peripheral TRPV1 in prolonged or chronic inflammatory pain, we previously reported that TRPV1, detected with immunohistochemical staining, was increased in DRG for over 28 days following CFA injection [16]. Thermal hyperalgesia persisted from day 1 to day 28. With area frequency analysis, we also reported that there was a shift of TRPV1 expression from small to medium-sized neurons.

In the present study, we continue our efforts in clarifying the role of TRPV1 in DRG in chronic inflammatory pain. Using the rat model of CFA-induced hypersensitivity, we examined TRPV1 expression more precisely with Western blotting and observed TRPV1 expression in three different subtypes of DRG neurons with double staining of neuronal markers. In addition, the TRPV1 antagonist AMG 9810 was injected intrathecally to prove the role of TRPV1 in thermal hyperalgesia as well as in mechanical allodynia.

## Methods

### Animals

Male Sprague-Dawley rats (200~300 g) were housed under diurnal light-dark cycles and provided water and food *ad libitum*. Rats were habituated to the testing paradigms for 3~5 days before data collection. All protocols were approved by the Animal Care and Use Committee of Peking University Health Science Center, and followed the Guidelines of Animal Use and Protection in our university adopted from the National Institutes of Health Guide for the Care and Use of Laboratory Animals (NIH Publications No. 80-23) revised in 1996. All possible efforts were made to minimize unnecessary suffering of animals.

### CFA inflammatory pain model

One hundred μl of CFA (Sigma-Aldrich, St. Louis, USA) was injected into the plantar surface of the left hind paw or the rats to induce inflammatory hyperalgesia [17]. Classical signs of acute inflammation including edema, redness and heat were most intense from day 1 to day 3 after injection, and lasted more than 4 weeks. Normal saline was similarly injected in the hindpaw of rats in the control group.

### Behavioral tests

Hot plate was used to test thermal hyperalgesia. Rats were habituated to the experimental environment for 30 min in their home cage. Rats were placed on a hot plate (52 ± 0.5°C) and the time until the rat jumped or licked either of its hind paws was recorded as hot plate latency (HPL). Following a response, the rat was immediately removed from the plate. Each testing was repeated three times with 15 min interval between tests. Latencies from the three tests were averaged.

Previous reports have shown that learning occurs during repeated exposure to a hot plate [18-20]. We also see that when hot plate latency was tested repetitively for 28 days, not only did CFA-injected rats show obvious hyperalgesia but also control rats (data not shown). Thus, we used two groups of rats at each time point, one was the saline-injected control group, and the other was CFA-injected group. HPL was measured before CFA injection or before saline injection and used as basal HPL, it was again measured and recorded at each time point. Percentage change of HPL was calculated as follows: % change = (basal HPL - HPL)/basal HPL × 100%.

Von Frey hair test was carried out according to the method described by Chaplan et al. [21] and modified by Dixon to measure mechanical allodynia. Each animal was placed in clear plexiglas compartment with a mesh floor and was allowed to habituate for 20 min. At days 1, 3, 7, 14, 21 and 28 after CFA injection, mechanical allodynia was evaluated with von Frey hair (Semmes-Weinstein Monofilaments, North Coast Medial Inc., San Jose, CA) in ascending order of force (0.41~15.1 g) to the plantar surface of the hind paw. Two groups (control and CFA) animals were used as in the above-mentioned HPL test at each time point.

Paw withdrawal threshold (PWT) was measured before CFA or saline injection and it was used as basal PWT. PWT was measured again at each time point after CFA or saline injection. In addition, percentage change of PWT was calculated as follows: % change = (basal PWT - PWT)/basal PWT × 100. PWT was measured before CFA injection and on days 1, 3, 7, 14, 21 or 28 after CFA injection.

### Application of AMG 9810

Five days before setting up CFA model, a PE10 tube was surgically placed into the subarachnoid cavity. AMG 9810 (Todris Cookson, Bristol, UK) was administered in 5% ethanol/95% saline in a volume of 10 μl. To examine the possible inhibition on mechanical and thermal hyperalgesia, AMG 9810 (5, 15, 45 μg) or its vehicle was administrated intrathecally on day 7. HPL or PWT was measured before AMG 9810 or vehicle administration, and 1 h, 2 h, 4 h, 24 h, 48 h after administration. Reversal of thermal hyperalgesia or mechanical allodynia was calculated according to the following formula: % of reversal = (post-HPL or PWT - pre-HPL or PWT)/(base threshold - pre-HPL or PWT) × 100%.

### TRPV1 expression detection with Western blot

Western blot was performed to measure TRPV1 expression on days 1, 3, 7, 14, 21 and 28 in CFA group, and on day 14 in control (naïve) group (n = 3/group). L4~L6 DRG were dissected. The tissue was homogenized in buffer containing 50 mmol/L Tris (pH 7.5), 250 mmol/L NaCl, 10 mmol/L EDTA (pH 8.0), 0.5% NP40 (Sigma, MO, USA), 10 μg/ml leupeptin (Sigma), 1 mmol/L PMSF (Sigma), 4 mmol/L NaF. The supernatants were centrifuged at 1000 g for 10 min at 4°C. The protein concentration was determined with a protein-quantification kit (Pierce, USA). Protein samples (20 μg/well) were separated through sodium dodecyl sulphate polyacrylamide (Bio-Rad, Hercules, CA) gel electrophoresis (10% gel) and then transferred to polycinylidene difluoride filters (Bio-Rad). The blots were blocked by 5% milk for 60 min at room temperature, and incubated with anti-TRPV1 primary antibody (1:500, Calbiochem, Oncogene, USA) for more than 48 h at 4°C. The blots were then incubated in horseradish peroxidase (HRP)-conjugated anti-rabbit secondary antibody (1:2000, Jackson, USA) for 60 min at room temperature, and developed in ECL™ (enhanced chemiluminescence) solution (Santa Cruz, USA). The standardization ratio of TRPV1 to β-actin band density was used to calculate the change in TRPV1 expression.

### Double immunofluorescent staining of TRPV1 with DRG neuronal markers

Rats were over-anesthetized and then transcardially perfused with saline and 4% paraformaldehyde (pH 7.4) sequentially. The left L4 DRG were quickly removed, post-fixed with paraformaldehyde, and then places in a sucrose solution. Sections of 8 μm in thickness were cut along DRG long axis on a cryostat and mounted on 3-aminopropyl-triethoxysilane-coated glass slides. There was 8. μm distance between sections. For each DRG, three sections at 1/4, 1/2, 3/4 positions were chosen from 3 rats were measured at each time point. DRG sections were co-incubated with a combination of rabbit anti-rat TRPV1 antibody (1:200, Calbiochem, Oncogene, USA) in 1% bovine serum albumin and 0.3% Triton-X100 in 0.01 mol/L phosphate-buffered saline (PBS) and one of the following antibodies: (1) mouse anti-rat neurofilament 200 (NF200) (1:1,000, Sigma), (2) fluorescein isothiocyanate-labeled isolectin B4 (IB4-FITC; 10 μg/ml, Sigma), (3) mouse anti-rat calcitonin gene-related peptide (CGRP, 1:1,000, Sigma) over night at 4°C. After washing with phosphate-buffered saline, sections were incubated with rhodamine isothiocyanate (TRITC)-conjugated affinipure goat anti-rabbit IgG (H+L) (1:2000, Jackson, USA) and fluorescein isothiocyanate-conjugated goat anti-mouse IgG (H+L) (1:2000, Jackson) except for IB4 for 40 min at 37°C. Stained slides were viewed and photographed with a CCD camera under a fluorescent microscope (DMIRB, Leica, Germany).

To further analyze whether there was a shift of TRPV1 expression from small neurons to myelinated A fiber neurons, area frequency of TRPV1 positive neurons in NF200 positive neurons was analyzed. In both control and CFA-induced inflammatory animals, 850 to 1500 DRG neurons with TRPV1 and NF200 double positive staining at day 3 were analyzed as an example. TRPV1 positive neurons in NF200 positive neurons of different sizes were allocated to separate bins and plotted against the percentage of total DRG neurons analyzed. In order to minimize statistical error, only the cell with a clear nucleus was selected. Because CGRP or IB4 stained all small DRG neurons, it was not necessary to analyze area frequency distribution in neurons of TRPV1 double staining with CGRP or IB4.

### Statistical analysis

All statistical analyses were performed using two-way analysis of variance (ANOVA) followed by Bonferroni *post-hoc *test, except Western blot was analyzed with one-way ANOVA followed by Newman Keuls *post-hoc *test. Statistical significance was determined as p < 0.05.

## Results

### Thermal hyperalgesia and mechanical allodynia following CFA injection

Time course of thermal hyperalgesia after CFA injection showed that the HPL and the percent change of HPL decreased significantly in the CFA group at day 1 to day 28 with the shortest at day 7. On day 7, the HPL was 4.8 ± 0.3 s in the CFA group and 9.5 ± 0.5 s in the control group; percent change of HPL was 59.7 ± 3.1% in the CFA rats, and 17.9 ± 4.3% in the control rats.

For mechanical allodynia, it was found that PWT and percent change of PWT decreased from day 1 to day 14, especially at days 1, 3 and 7. For example, on day 7, the PWT was 2.0 ± 0.3 g in the CFA group and 9.7 ± 1.0 g in the control group. Percent change of PWT increased from day 1 to day 14 significantly (p < 0.001). For example, at day 7, it was 84.0 ± 2.7% in the CFA group and 18.3 ± 6.3% in the control group respectively.

### Antagonizing effect of AMG 9810 on thermal hyperalgesia and mechanical allodynia

**Figure 1:**
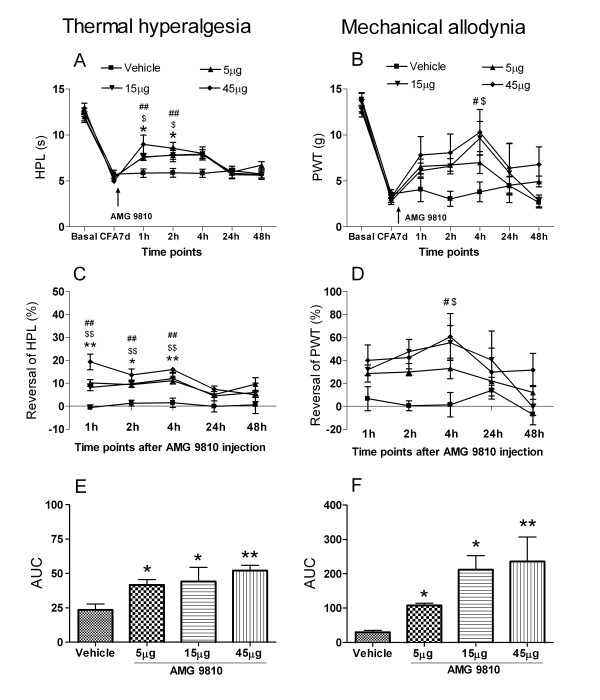
**Antagonizing effect of AMG 9810, a specific TRPV1 antagonist, on thermal hyperalgesia and mechanical allodynia on day 7 after CFA injection. A**, **C**, and **E **in left panel for thermal hyperalgesia presented by hot plate latency (HPL); **B**, **D **and **F **in right panel for mechanical allodynia presented by paw withdrawal latency (PWT). **A**, Hot plate latency (HPL); **C**, Reversal of HPL (%); **E**, Area under the curve (AUC) of percentage of reversal of HPL. **B**, Paw withdrawal threshold; **D**, % Reversal of PWT; **F**, Area under the curve (AUC) of percentage of reversal of PWT. After AMG 9810 application, HPL and % reversal of HPL significantly increased at doses of 5 to 45 μg (p < 0.05, 0.01 or 0.001) from 1 h to 4 h; PWT and the reversal of PWT (%) displayed similar results, the reversal of HPL (%) significantly increased at doses of 5 to 45 μg (p < 0.05 or 0.01) at 4 h. *, $ and # represent comparison of AMG 9810 at 5, 15 or 45 μg with vehicle, respectively. n = 5–13.

Intrathecal injection of AMG 9810 significantly reduced thermal allodynia (Fig. 1A and 1C) and mechanical hyperalgesia (Fig. 1B and 1D). After administration, AMG 9810 at all three doses of 5, 15 and 45 μg per animal significantly reduced thermal hyperalgesia at 1 h, 2 h and 4 h (p < 0.05). The HPL values of AMG 9810 at three doses at 1 h were 7.6 ± 0.3 s, 7.6 ± 0.4 s and 9.0 ± 1.0 s, while HPL of vehicle was 5.9 ± 0.4 s (Fig. 1C). The average percent changes of AMG 9810 at three doses at 1 h were 9.3 ± 2.0%, 10.2 ± 1.8% and 10.3 ± 3.0%, respectively; while that of the vehicle was 0.5 ± 1.2%. Area under the curve (AUC) analysis of the percent change of HPL found that the AMG 9810 effect was dose-dependent (Fig. 1E).

AMG 9810 also reduced mechanical allodynia from 1 h to 24 h after application, and the effect lasted up to 48 h following the 45 μg dose. The PWT values of AMG 9810 at the three doses at 4 h were 7.0 ± 1.2 g, 9.6 ± 1.8 g or 10.3 ± 2.5 g, respectively, while that of the vehicle group was 3.8 ± 1.1 g (Fig. 1B). The average percent changes of AMG 9810 at 4 h at doses of 5, 15 and 45 μg were 33.0 ± 9.0%, 55.4 ± 14.9% and 60.6 ± 20.4%, respectively; while that of vehicle was 1.5 ± 10.6%. There was significant difference between 5 μg, 15 μg, 45 μg groups and vehicle group at 4 h (Fig. 1D) (p < 0.05). From AUC analysis of the percent change of PWT, it was found that the effect of AMG 9810 was dose-dependent (Fig. 1F).

### TRPV1 protein expression increase in DRG

**Figure 2:**
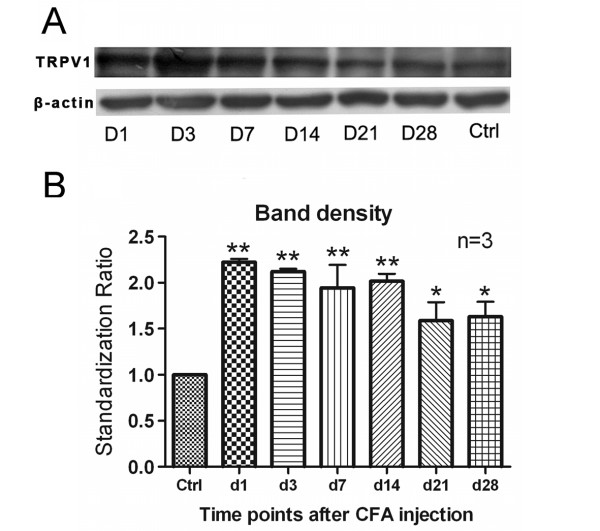
**Western blotting detection of TRPV1 protein in L4~L6 DRG. A**, Western blotting bands. Upper row: TRPV1; Lower row: β-actin. **B**, Optical band density analysis. TRPV1 protein increased significantly after CFA injection from day 1 to day 28 (* p < 0.05). n = 3.

TRPV1 protein expression was measured with Western blotting in L4~L6 DRG 1, 3, 7, 14, 21, and 28 days after CFA injection. A representative result is shown in Fig. 2A and the standardization ratio statistics of three tests is shown in Fig. 2B. The average intensities of the bands in CFA group increased significantly from day 1 to day 28. When the density in the control group was standardized to 1.0, the average densities were 2.23 ± 0.04, 1.94 ± 0.25 and 1.63 ± 0.16 at days 1, 7 and 28 after CFA injection, suggesting that TRPV1 protein increased in DRG.

### TRPV1 distribution change in different subtypes of DRG neurons

**Figure 3:**
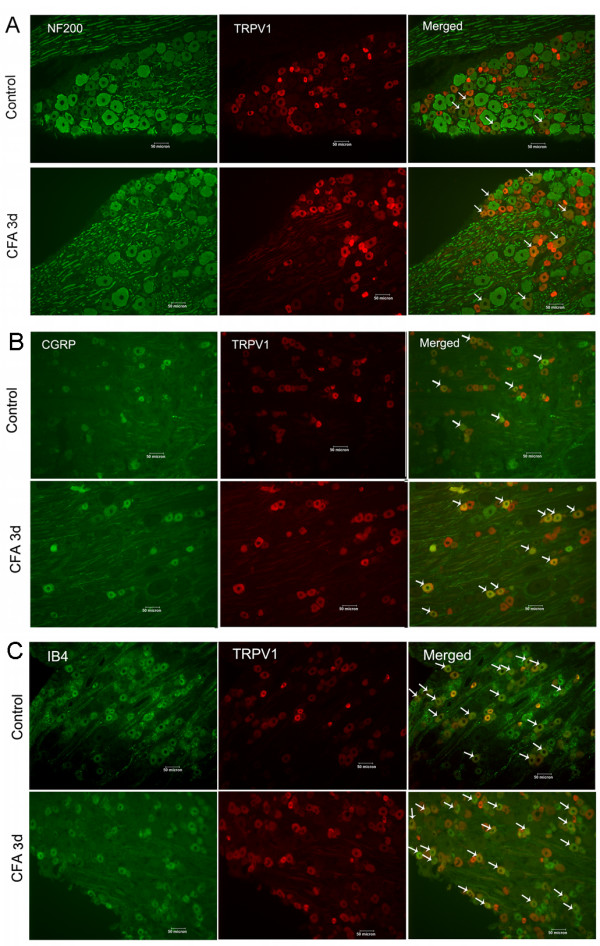
**Double immunofluorescent staining of TRPV1 in DRG with neuronal markers of three subtypes at day 3 as an example**. **A**, with NF200. In control animals, rare double staining of TRPV1 with NF200 was seen; after CFA injection, double staining of TRPV1 with NF200 significantly increased. **B**, with CGRP. Double staining of TRPV1 with CGRP significantly increased in CFA rats. **C**, with IB4. In control animals, double staining of TRPV1 with IB4 was ~50%; after CFA injection, double staining with IB4 significantly increased. Arrows indicate the doubly-labeled staining. Scale bar: 50 μm.

**Figure 4:**
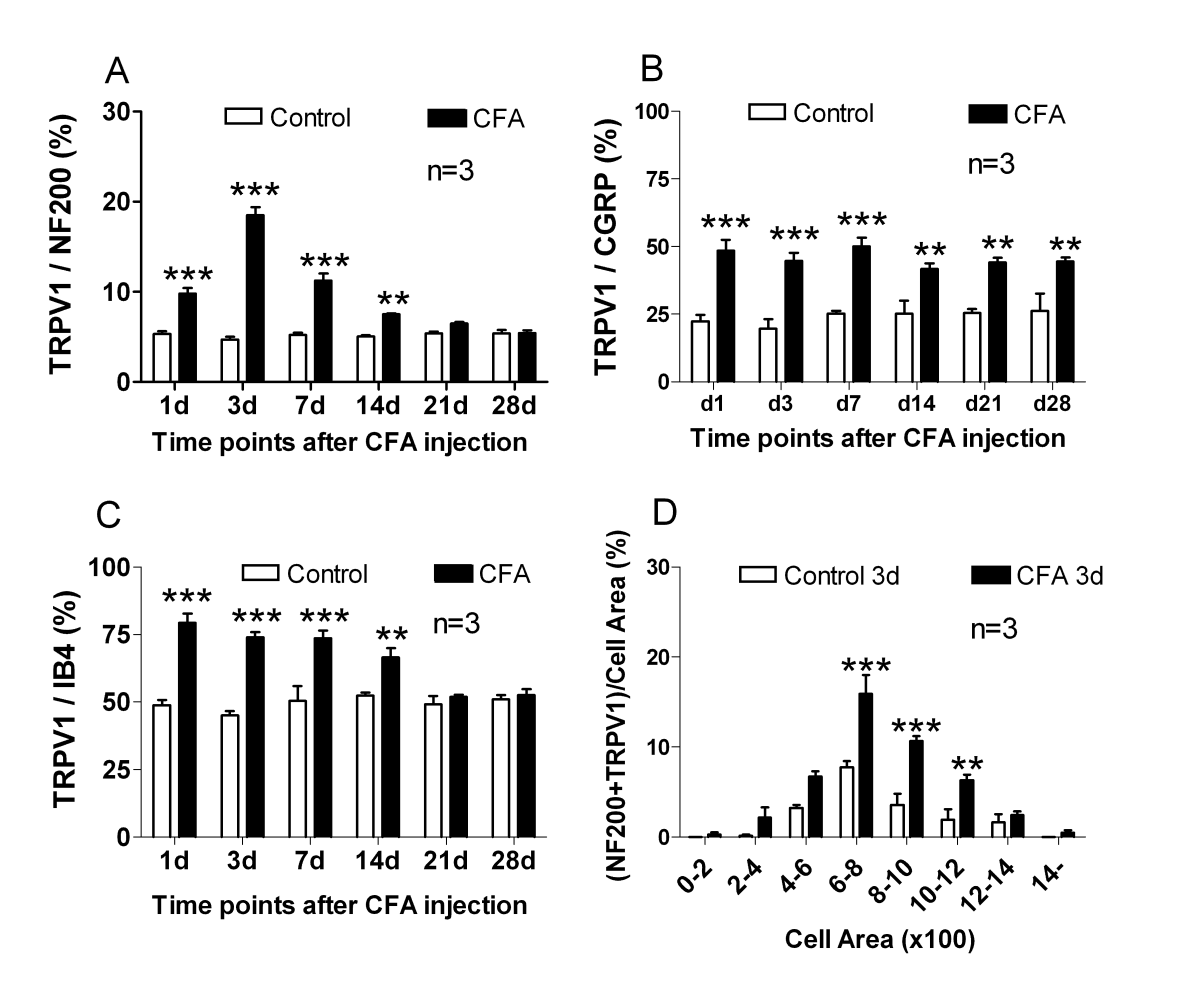
**Quantification analysis of DRG neurons doubly stained with TRPV1 and neuronal markers**. **A**, **B **and **C **show the percentage of TRPV1 positive neurons to NF200-, CGRP-, IB4-positive neurons, respectively. **A**, In control animals, rare double staining with NF200 was seen; after CFA injection, double staining of TRPV1 with NF200 significantly increased at days 1, 3, 7 and 14. **B**, Double staining of TRPV1 with CGRP significantly increased in CFA rats from day 1 to day 28. **C**, In control animals, double staining of TRPV1 with IB4 was ~50%, significantly increased from day 1 to day 14 after CFA injection. **D**, Area frequency distribution of TRPV1 and NF200 double stained neurons at day 3. After CFA injection, double staining neurons significantly increased in the medium-sized neurons with cell area of 600–1200 μm^2^. * p < 0.05, ** p < 0.01, *** p < 0.001. n = 3.

To examine in which subtypes of DRG neurons TRPV1 expression was increased, TRPV1 was doubly stained with three neuronal makers NF200, IB4 and CGRP. Anti-NF200 antibody recognizes high molecular weight neurofilaments and is used as a marker of myelinated A fiber neurons including large and the medium-sized ones. In the control animals, only a few (5.3 ± 0.3%) NF200-positive neurons expressed TRPV1, but after CFA injection, the percentage increased significantly from day 1 till day 14, especially at day 3 (18.5 ± 0.9%) (p < 0.001) (Fig. 3A and Fig. 4A).

Small DRG neurons can be neurochemically divided into two subtypes: the peptidergic neurons marked by CGRP as a marker and the non-peptidergic ones marked by IB4. For CGRP-positive peptidergic neurons, in the control animals, small amount (22.3 ± 2.4%) expressed TRPV1; in the CFA rats, the percentage increased significantly from day 1 to day 28. The percentages were 48.5 ± 4.0%, 50.0 ± 3.2% and 44.5 ± 1.4% at day 1, day 7 and day 28, respectively (p < 0.001) (Fig. 3B and Fig. 4B). For IB4-positive non-peptidergic neurons, in the control animals, about half (48.9 ± 1.9%) expressed TRPV1, while in the CFA rats, the doubly stained neurons increased significantly from day 1 to day 14, the percentages were 76.2 ± 2.0%, 74.0 ± 1.9%, 73.7 ± 2.8% and 66.5 ± 3.4% respectively (p < 0.001) (Fig. 3C and Fig. 4C).

Area frequency histograms showed that TRPV1 expression in NF200 positive DRG neurons in the control animals was mainly within small neurons (cell area < 800 μm^2^) (Fig. 4D). The ratio of TRPV1 and NF200 doubly stained neurons at 3 d in the CFA rats increased mainly within neurons with cell area of 600–1200 μm^2^, which were mainly the medium-sized A-fiber neurons. Within neurons with cell area of 600–800, 800–1000 and 1000–1200 μm^2^, the percentages of TRPV1 and NF200 doubly stained neurons in the control group were 7.7 ± 0.7%, 3.6 ± 1.3% and 1.9 ± 1.2%, respectively; while in the CFA group, the percentages increased to 15.9 ± 2.1%, 10.7 ± 0.6% and 6.3 ± 0.6%, respectively (p < 0.01 or p < 0.001).

## Discussion

### Thermal hyperalgesia and mechanical allodynia during chronic inflammatory pain

It is well known that thermal hyperalgesia occurs within several days after CFA injection, *i.e*. in the acute phase of inflammatory pain. In recent years, some researchers found that there was a mechanical allodynia in acute inflammatory pain rats. [22,23]. The previous study all observed the acute phase inflammation. In the present study, we observed that thermal hyperalgesia lasted from day 1 to day 28 in the CFA inflammatory rats relative to the control rats which was consistent with our previous report [16], but in a more strict way to avoid repeated effects. The study not only confirmed that 100% CFA could induce a one month long inflammation with strong thermal hyperalgesia but also observed mechanical allodynia in a longer period–one month and proved mechanical allodynia appeared chronically following CFA injection from day 1 to day 7 not only appeared in the acute phase.

### Involvement of TRPV1 in thermal hyperalgesia and in mechanical allodynia during chronic inflammatory pain

In our previous study [16], TRPV1 expression was detected with grey density analysis of DRG immunohistochemical staining, a semi-quantification method. In the present study, with Western blotting, a quantification method, we confirmed our previous report with immunohistochemical staining that TRPV1 protein expression in DRG was significantly increased relative to control beginning day 1 and throughout the 28 after CFA injection (Figs. 2, 3 and 4).

TRPV1 is speculated to play a role in the development of thermal hyperalgesia under chronic inflammatory pain according to the changes of its expression and behaviorally thermal hyperalgesia. In the present study, thermal hyperalgesia and the expression of TRPV1 also correlates well (data not shown). Our previous report could give only weak evidence. In the present study, with the additional reversal using the TRPV1 antagonist AMG 9810 [15], we provide direct evidence for the role of TRPV1. It was found that AMG 9810 could reverse thermal hyperalgesia with a dose-dependent manner (Fig. 1). These results together with the pharmacological results of the TRPV1 antagonist AMG 9810 (Fig. 1) provide direct evidence that TRPV1 plays a role in thermal hyperalgesia in a relatively long period in CFA inflammatory pain.

More interestingly, with this same paradigm, we found that TRPV1 also plays a role in mechanical allodynia. For mechanical allodynia, although TRPV1 expression was elevated during the 28 days after CFA injection (Fig. 2), the mechanical allodynia was only increased significantly from day 1 to day 14, with peak at day 7 (Data not shown). The antagonist reversal (Fig. 1D and 1F) provides strong evidence that the increased TRPV1 participated in mechanical allodynia during chronic inflammatory pain. Cui *et al*. reported that TRPV1 might play roles in acute CFA inflammatory pain. Two TRPV1 antagonists (with good or poor CNS penetration) could reduce mechanical allodynia and thermal hyperalgesia on day 2 after 50% CFA injection. In the present study, a longer period after 100% CFA injection was observed. TRPV1 antagonist – AMG 9810 could reduce thermal hyperalgesia as well as mechanical allodynia at a longer time point – day 7. We could choose a much longer time point to do the pharmacology test. We chose day 7, because at this time point thermal hyperalgesia and mechanical allodynia reached their highest level.

The results showed that AMG 9810 had a long-term effect on mechanical allodynia compared to thermal hyperalgesia (Fig. 1). AMG 9810 reduced thermal hyperalgesia at 1 h, 2 h and 4 h; it reduced mechanical allodynia from 1 h to 24 h after application and the effect lasted up to 48 h at dose of 45 μg. It is an interesting phenomenon and it seems to suggest that TRPV1 has differential role in mechanical allodynia and in thermal hyperalgesia.

What we should notice is that there was a difference in the time courses of TRPV1 protein expression and of thermal hyperalgesia or mechanical allodynia. Taking thermal hyperalgesia for instance, HPL was lowest at day 7 (data not shown), while TRPV1 reached its peak at day 1 through day 14 (Fig. 2). This discrepancy may indicate that TRPV1 is not the only molecule to determine thermal hyperalgesia, and possibly there are other ion channels or receptors involved.

### Shifting and role of the increased TRPV1 expression in different subtypes of DRG neurons during chronic inflammatory nociception after CFA injection

The present study showed that TRPV1 protein in DRG increased from day 1 to day 28 after CFA injection (Fig. 2). One more interesting question is which subtype of DRG neurons the increased TRPV1 is located. It is well known that different subtypes of DRG neurons have different functions. In our previous report [16], with area frequency distribution analysis, we found a shift of TRPV1 expression from small to medium-sized DRG neurons over the observation period of 28 days. But we did not know exactly the nature of neurons with different size in our previous report.

In the present study, with specific neuronal markers, we demonstrated more clearly the up-regulation of TRPV1 expression in specific subtypes of DRG neurons in CFA inflammatory rats. We found that TRPV1 expression increased in distinct small DRG neurons. As shown in Fig. 4, TRPV1 increased within CGRP positive neurons from day 1 to day 28 (Fig. 3B, 4B), within IB4 positive neurons from day 1 to day 14 (Fig. 3C, 4C), most of which were C-fiber neurons [24]. TRPV1 was more doubly stained with NF200, and the ratio of the doubly-labeled neurons significantly increased from day 1 to day 14 (Fig. 3A, 4A), suggesting that more myelinated A fiber neurons expressed TRPV1. So, in the early stage (from day 1 to day 14 as in the present study) after CFA injection, TRPV1 in all three subtypes of DRG neurons might participate in the inflammatory hypersensitivity, while in the late stage (from day 21 to day 28), only TRPV1 in CGRP positive neurons might take place. We may propose that C-fiber neurons play more roles in mechanical allodynia since CGRP and TRPV1, IB4 and TRPV1 double stained neurons increased at day 7; mechanical allodynia reached its peak at day 7 and could be reversed by TRPV1 antagonist.

Interestingly, NF200 positive neurons expressed more TRPV1 after CFA injection. These neurons were mainly the medium-sized (600–1200 mm^2^) (Fig. 4D). This result clearly demonstrates that the medium-sized neurons expressing TRPV1 include myelinated, A-delta fiber neurons. The shifting of TRPV1 expression to the myelinated neurons occurred not only in the acute phase immediately after CFA injection, but also in the chronic stage of inflammatory pain as shown in the present study. Similar shifting has also been reported in the diabetic neuropathy model and bone cancer pain model animals [25-27].

Some studies reported a strong memory process in hot plate testing [18-20]. We also found that repetitive measurement paradigm induced memory on the hot plate test (data not shown) and in the von Frey hair test. In our previous study [16], we, like many other researchers, routinely tested one group of animals repetitively at different time points. There should exist possible learning and memory effects. In order to avoid this, in the present study, we used two parallel groups of rats at each time point throughout the entire 28-days observation. With this more strict design, the evidence should be more reliable for the role of TRPV1 in thermal hyperalgesia and mechanical allodynia in chronic inflammatory pain.

## Conclusion

In summary, with Western blot and behavioral pharmacological methods, we give further strong evidence for the role of TRPV1 in CFA-induced chronic inflammatory pain including thermal hyperalgesia and mechanical allodynia. TRPV1 in distinct subtypes of DRG neurons plays a role not only in the acute, but also in the chronic inflammatory pain. Mechanical allodynia exists in chronic inflammatory pain, in which TRPV1 may also take effect.

## Abbreviations

AUC: area under the curve; CFA: complete Freund's adjuvant; CGRP: calcitonin gene-related peptide; DRG: dorsal root ganglion; HPL: hot plate latency; IB4: isolectin B4; NF200: neurofilament 200; PWT: paw withdrawal threshold; TRPV1: transient receptor potential vanilloid 1.

## Competing interests

The authors declare that they have no competing interests.

## Authors' contributions

LY participated in the design of the study, carried out all the experiments, performed the statistical analysis and drafted the manuscript. FY contributed as co-first author, designed and took part in the behavior tests. HL and FYL participated in the design of the study. YW, GGX and JSH participated in the design of the study, assisted with the data analysis and interpretation and wrote the manuscript. LY and FY contributed equally. All authors have read and approved the final manuscript.
